# Comprehensive microRNA analysis toward exploring a new functional component in Matcha green tea

**DOI:** 10.1016/j.fochms.2025.100265

**Published:** 2025-05-28

**Authors:** Yi-Lan Huang, Tomomi Morikawa-Ichinose, Seong-Uk Lee, Yuka Tatsumi, Masaki Ichitani, Motofumi Kumazoe, Hirofumi Tachibana, Yoshinori Fujimura

**Affiliations:** a Division of Applied Biological Chemistry, Department of Bioscience and Biotechnology, Faculty of Agriculture, Kyushu University, 744 Motooka, Nishi-ku, Fukuoka 819-0395, Japan; b Central Research Institute, ITOEN, Ltd., 21 Mekami, Makinohara-shi, Shizuoka 421-0516, Japan

**Keywords:** Green tea, Matcha, Plant-based dried powders, MicroRNA, Chemical composition, Extraction methods

## Abstract

Matcha, a traditional Japanese green tea, has health-promoting effects. However, little is known about its bioactive components, except for polyphenols, caffeine, and amino acids. Here, we revealed the presence of diverse miRNAs, a type of functional RNAs, as new components of Matcha, using next-generation sequencing. Quantitative reverse-transcription PCR analysis of 10 different Matcha showed that miRNA levels varied depending on the cultivar and harvest season. As extraction methods of miRNAs, we found that soaking at 95 °C significantly enhanced total RNA and miRNA yields. Furthermore, a positive correlation was observed between total RNA and miRNA yields extracted from 27 plant-based dried powders. Notably, Matcha exhibited the highest levels of four representative miRNAs: lja-miR166-3p, csn-miR396d-5p, gma-miR396e, and csn-miRn409. The miRNA yields in Matcha were correlated with the major Matcha components. These results highlight Matcha as a source of miRNAs and candidate bioactive components. These findings provide new insights into the functionality of Matcha.

## Highlights

• Next-generation sequencing revealed the presence of diverse miRNAs in matcha.
• MiRNA levels in matcha differed depending on cultivars and harvesting seasons.
• Soaking matcha powders in 95 °C hot water enhanced RNA and miRNA yields.
• Matcha showed the highest levels of representative miRNA among plant-based powders.
• MiRNA yields in matcha were correlated with the major three matcha components.

## Graphical abstract

**Figure:**
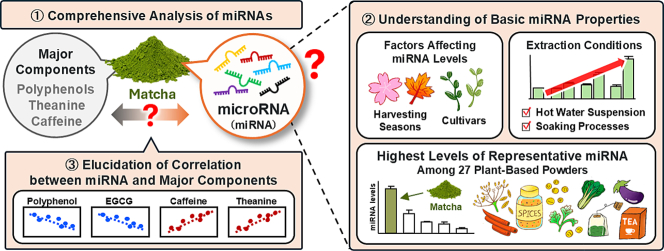

## 1 Introduction

Tea (*Camellia sinensis*), one of the oldest and most widely consumed beverages worldwide, comprises various components, including polysaccharides, amino acids, lipids, vitamins, and polyphenols (Sharangi, 2009). Teas are generally classified into several categories based on their manufacturing processes and degree of fermentation, including black, oolong, and green tea. Among them, green tea, an unfermented tea, has undergone extensive research demonstrating its various health-promoting effects, including anti-cancer (Kumazoe & Tachibana, 2016), anti-oxidative (Mokra et al., 2022), anti-inflammatory (Ohishi et al., 2016), and anti-allergic properties (Maeda-Yamamoto & Tachibana, 2020). The beneficial effects of green tea have been attributed to various bioactive compounds, including polyphenols (Kumazoe & Tachibana, 2016), caffeine (Westerterp-Plantenga, 2010), and amino acids (Williams et al., 2019). Among these, polyphenols have received the most attention and are responsible for a large portion of the physiological benefits of green tea (Musial et al., 2020; Tachibana, 2011; Umeda et al., 2008).

Matcha is a subset of Japanese green tea known for its unique cultivation methods, processing procedures, and consumption methods compared to sencha, the major type of green tea infusion. The major differences between matcha and sencha are the shading of tea plants during the growth period and the grinding of tea leaves into powder using stone mills. This special cultivation method enhances the synthesis of bioactive compounds, including caffeine, theanine, chlorophyll, and various types of catechins, resulting in the accumulation of higher concentrations of these components in Matcha (Devkota et al., 2021; Jakubczyk et al., 2020; Kochman et al., 2020). Grinding Matcha tea leaves into a fine powder allows the entire powder, not just the extract, to be consumed. This enables both the water-soluble and water-insoluble components in Matcha to be fully ingested and utilized, thereby enhancing their health-promoting potential (Fujioka et al., 2016; Zhou et al., 2021). Moreover, the powdered form of Matcha allows it to be consumed not only as a hot water infusion but also as a food additive in various snacks and food products. By contrast, sencha is mostly consumed as an infusion (Devkota et al., 2021). Various consumption methods have contributed to the increasing global popularity and market demand for Matcha (Devkota et al., 2021).

Despite its distinctive characteristics, Matcha contains biologically important molecules similar to sencha, including polyphenols, amino acids, caffeine, and vitamins (Horie et al., 2017). Consequently, Matcha has received considerable attention for its health-promoting effects, such as antioxidant, anti-inflammatory, and antibacterial properties (Burcuș et al., 2018), as well as its anti-cancer (Bonuccelli et al., 2018), anti-obesity (Y. Wang et al., 2022), and stress-reducing effects (Unno et al., 2018). Although some studies suggested these effects could be attributed to the main bioactive compounds, such as catechins and theanine (Unno et al., 2018), functionality research on the components, except for these two main bioactive compounds, is still limited. This raises the question of whether other functional components contribute to the physiological effects of Matcha.

In biomedical and food functionality research, microRNAs (miRNAs, miRs), a class of single-stranded non-coding RNA, approximately 22 bases in length, have received considerable attention. miRNAs negatively regulate gene expressions by complementarily binding to 3′-untranslated regions of their target messenger RNA (mRNA) (Ge et al., 2018), and miRNAs are estimated to be involved in regulating 60 % of genes in animals (Friedman et al., 2009). Thus, it is generally known that miRNAs play a crucial role in various biological processes, particularly in life phenomena and disease control (Ardekani & Naeini, 2010; Yamada et al., 2015).

Recent studies suggest that plant-derived miRNAs have exert beneficial effects (Chen et al., 2020; Kumazoe et al., 2023; Liu et al., 2021; Samad et al., 2021; L. Zhang et al., 2011, Zhang et al., 2019). Compared with animal miRNAs, plant miRNAs are robust due to the methylation of the second hydroxyl group of ribose at the 3′ end (X. Wang et al., 2020). This robustness of plant miRNAs allows them to be stably absorbed into the circulatory system and organs, where they regulate gene expression and mediate various biological phenomena (Liang et al., 2015; X. Wang et al., 2020). Recently, an in vivo animal model demonstrated that miR172d-5p, a plant miRNA derived from rice (*Oryza sativa*), exerted antifibrotic effects by suppressing the expression of fibrosis-related genes (Kumazoe et al., 2023). Similarly, studies have shown that miR159a, derived from soybeans, inhibits colon tumorigenesis (Liu et al., 2021) and miR2911, derived from honeysuckle, which is often consumed as an herbal tea in China, inhibits liver fibrosis in mice (Chen et al., 2020). Several studies have demonstrated the remarkable potential of food-derived plant miRNAs to have various biological implications in animals through cross-kingdom regulation (Samad et al., 2021; L. Zhang et al., 2019).

Recently, miRNAs in tea plants have been investigated using both computational identification (Prabu & Mandal, 2010; Zhu & Luo, 2013) and experimental methods, such as high-throughput small RNA sequencing (Jeyaraj et al., 2017; Zheng et al., 2015). These studies have often utilized fresh leaves of tea plants as samples and investigated plant biology, including the assessment of miRNA responses to environmental stress (Jeyaraj et al., 2017; Zheng et al., 2015). By contrast, from the perspective of food science, instead of consuming fresh leaves directly, green tea is generally consumed as an infusion of dried leaves, which undergo various manufacturing processes, including steaming and drying. However, little is known regarding the presence of miRNAs and their physiological roles in Matcha, a representative Japanese green tea powder.

In this study, we aimed to analyze the presence of miRNAs in Matcha to explore new functional food factors and evaluate the effects of cultivars and harvest seasons. Moreover, to provide fundamental information for further functional analysis of miRNAs, we investigated the basic properties of miRNAs, including the evaluation of extraction methods and the relationship between miRNAs and total RNA or major functional compounds in Matcha.

## 2 Materials and methods

### 2.1 Materials

Ten types of Matcha powders with different cultivars and harvesting seasons were provided by ITOEN, Ltd. (Tokyo, Japan), including three spring-harvested cultivars called “Saemidori” (SMD), “Okumidori” (OM), “Samidori” (SM) as well as a blended Matcha (MIX), and seven samples harvested in autumn-winter seasons (“Saemidori” (SMD), “Okumidori” (OM), “Yabukita” (YB), “Yutakamidori” (YM), “Sayamakaori” (SK), “Kurasawa” (KS), “Zairai” (ZR)). Matcha samples were kept at −30 °C until RNA extraction. In addition to the abovementioned Matcha samples, 15 commercially available Matcha powders were purchased from local shops in Japan and stored at room temperature (RT). Before RNA extraction, 30 mg of Matcha powder was suspended in 1 mL of 80 °C nuclease-free water (NFW; Ambion, MA, USA). To evaluate the extraction methods, 30 mg of Matcha powder was suspended in NFW at different temperatures and immediately incubated at the same temperature using a CTU-Neo Cool Thermo Unit (TAITEC, CA, USA) before RNA extraction.

Twenty-six plant-based dried powdered products (excluding Matcha powder) were purchased from a local supermarket in Fukuoka-shi, Japan and stored at RT. Before analysis, non-powdered samples were homogenized using stainless steel beads (stainless steel φ4.8 mm, TOMY, Tokyo, Japan) with a Micro Smash MS-100 Cell Disruptor (TOMY) at 3500 rpm, 4 °C, for three cycles of 30 s.

### 2.2 Small RNA extraction for sequencing

Small RNAs were extracted from the Matcha powder using a combination of QIAGEN columns. In brief, 44 mg of the Matcha sample was suspended in 900 μL of 80 °C nuclease-free water (Ambion, MA, USA). To 90 μL of the suspension solution, 900 μL of QIAzol Lysis Reagent (QIAGEN, Hilden, Germany) was mixed and transferred to the QIAGEN Shredder Spin Column (QIAshredder, QIAGEN). After centrifugation (20,000*g*, 4 °C for 2 min), the flow-through was added to 198 μL of chloroform (CHCl₃), followed by incubation at RT for 3 min. After centrifugation (15,000*g*, 4 °C for 15 min), 350 μL of the water layer was collected and added to 350 μL of 70 % ethanol. The sample was then transferred to the RNeasy Mini Spin Column (RNeasy Mini Kit, QIAGEN) and centrifuged (8000*g*, RT for 15 s). The flow-through was added to 400 μL of 100 % ethanol and then transferred to the Mini Elute Column (QIAGEN, Hilden, Germany). After centrifugation (8000*g*, RT for 15 s), the flow-through was discarded, and 700 μL of RWT buffer was added to the column. Centrifugation (8000*g*, RT for 15 s) was then carried out, and 500 μL of RPE buffer (RNeasy Mini Kit, QIAGEN) was added to the column after discarding the flow-through. Then, the column was centrifuged (8000*g*, RT for 15 s), and 500 μL of 80 % ethanol was added after discarding the flow-through. After centrifugation (8000*g* at room temperature for 2 min), the column was transferred to a new tube and centrifuged (8000*g* for 5 min). Then, the column was transferred to a new tube, and 14 μL of RNase-Free Water (RNeasy Mini Kit, QIAGEN) was added, followed by centrifugation (8000*g*, RT for 1 min). Finally, the flow-through was collected, and the RNA concentration was measured using a NanoDrop 2000 (Thermo Fisher Scientific, MA, USA).

### 2.3 Small RNA library preparation and sequencing

To construct a sequencing library from the extracted small RNA, we used the TruSeq Small RNA Set A MiniSeq Kit (Illumina, California, USA) according to the manufacturer's protocol. Briefly, small RNAs (sRNAs) were ligated to 3′ and 5′ RNA adapters using T4 RNA Ligase 2 truncated (New England Biolabs, MA, USA). The ligated products were then reverse-transcribed into complementary DNA (cDNA) using SuperScript II Reverse Transcriptase (Thermo Fisher Scientific, MA, USA). The cDNA was amplified by PCR using primers that were annealed to the ends of adapter. The sizes of the PCR products were determined using TBE gels (Bio-Rad, Hercules, CA, USA), and they were recovered by gel excision. The quality of the cDNA libraries was verified by determining their size and concentration using a high-sensitivity D1000 TapeStation (Agilent Technologies, Santa Clara, CA, USA) and Qubit 4 Fluorometer (Invitrogen, CA, USA), respectively. The samples were sequenced on an Illumina MiniSeq platform (Illumina, California, USA).

Data analysis was performed at Cell-Innovator (Fukuoka, Japan). miRNAs were aligned to all plant miRNAs available in the public miRNA database, miRBase (v21; http://mirbase.org/), as well as to *C. sinensis* miRNA sequences identified by Jeyaraj et al. (2017) with a mismatch of no more than two.

A heatmap of the sequenced data was generated using the statistical package MultiExperiment Viewer (MeV ver. 4.90) (https://mev.tm4.org/), followed by clustering analysis.

### 2.4 Total RNA extraction and quantification of Total RNA and miRNA yields

Total RNA was extracted from the Matcha samples using TRI reagent (Cosmo Bio Co., Tokyo, Japan) following the manufacturer's instructions. The concentration of the extracted RNA was determined using a NanoDrop 2000 spectrophotometer (Thermo Fisher Scientific, MA, USA) at absorbance wavelengths of 260 and 280 nm. miRNA concentrations were measured using a Qubit miRNA Assay Kit (Invitrogen, CA, USA) using a Qubit 4 fluorometer (Invitrogen, CA, USA) following the manufacturer's protocol.

### 2.5 Quantitative reverse-transcription PCR (qRT-PCR)

To detect plant miRNAs, we used the miRCURY LNA RT Kit (QIAGEN, Hilden, Germany) for reverse transcription of RNA to synthesize cDNA. qRT-PCR was conducted using a miRCURY LNA SYBR Green PCR Kit (QIAGEN, Hilden, Germany) on a CFX 96 Real-Time PCR System (Bio-Rad, Hercules, CA, USA) in accordance with the manufacturer's protocol. The primers for each miRNA (Table S2) were designed using “Custom miRCURY LNA miRNA PCR Assays” (QIAGEN, Hilden, Germany). Data were normalized to cel-miR39-3p (spike in control).

### 2.6 Evaluating Total polyphenol contents using the Folin–Ciocalteu method

The total phenolic content of the Matcha powder was measured using a modified version of the Folin–Ciocalteu method (Hovt et al., 1959). Powdered samples were diluted with ultrapure water to a 2.0 mg/mL concentration. Diluted samples (20 μL) were mixed with 100 μL of 0.2 N Folin and Ciocalteu's phenol reagent (Nacalai Tesque, Kyoto, Japan). Then, 80 μL of 40 % Na_2_CO_3_ solution (Nacalai Tesque, Kyoto, Japan) was added and incubated at RT for 60 min. Finally, absorbance was measured at 665 nm using an EnVision 2104 Multilabel Reader (PerkinElmer, Waltham, MA). The total polyphenol content of each sample was calculated using a calibration curve with gallic acid (Nacalai Tesque, Kyoto, Japan) as the standard.

### 2.7 Liquid chromatography-mass spectrometry (LC-MS) analysis

The contents of epigallocatechin-3-*O*-gallate (EGCG), theanine, and caffeine in Matcha powders were analyzed by a high-performance liquid chromatography (HPLC) system connected to a triple quadrupole-MS system (LCMS-8050, Shimadzu, Kyoto, Japan) equipped with a L-column 2 ODS (2.1 mm I.D. × 150 mm, 3 μm; CERI, Saitama, Japan) maintained at 40 °C. The mobile phase consisted of solvents A (H_2_O with 0.05 % formic acid) and B (0.05 % formic acid in acetonitrile). The gradient program was as follows: 0–1 min (A/B 90:10, *v*/v), 1–5 min (30:70, v/v), 5–7 min (0:100, v/v), 7–10.5 min (0:100 v/v), 10.5–11 min (90:10 v/v), and 11–15 min (90:10 v/v). The flow rate was 0.15 mL/min, and the injection volume was 10 μL. The ionization parameters included the nebulizer gas flow as 1.5 L/min, curved desorption line temperature as 250 °C, and heat block temperature as 400 °C. Compounds were detected using optimized multiple reaction-monitoring (MRM) transitions. The scores of the MRM transitions (precursor ion [*m/z*]/product ion [*m/z*]) for the three selected compounds were as follows: EGCG, 456.90/169.05 [M–H]^−^; theanine, 175.00/84.10 [M + H]^+^; and caffeine, 195.00/138.10 [M + H]^+^. Quality control (QC) samples, consisting of a mixture of all samples, were analyzed every 12 samples. The peak intensity of each compound was normalized using a QC-based correction, and the EGCG, theanine, and caffeine contents were calculated from a calibration curve using EGCG (Nagara Science, Gifu, Japan), L-theanine (FUJIFILM Wako Pure Chemical Industries Ltd., Osaka, Japan), and caffeine (Tokyo Chemical Industry Co., Ltd., Tokyo, Japan) as standards.

### 2.8 Statistical analysis

Data are presented as the mean ± standard error of the mean. Correlation analysis was performed using Pearson's correlation analysis. Prism software (GraphPad, Inc., San Diego, CA, USA) was used to perform statistical tests, including Student's *t*-test, Tukey's multiple comparison test, and Dunnett's comparison test. Statistical significance was set at *p* < 0.05.

## 3 Results

### 3.1 Comprehensive analysis of miRNAs in matcha

Plant-derived miRNAs, a type of functional RNA, have received considerable attention in food functionality research because of their potential health-promoting effects. However, no studies have investigated the presence of these miRNAs in Matcha. Here, we aimed to acquire the expression profiles of miRNAs in three types of Matcha, including two spring-harvested Matcha-orientated cultivars called “Okumidori (OM)” and “Samidori (SM)” as well as a blended Matcha (MIX). We conducted next-generation sequencing (NGS) analysis of hot water-extracted solutions of each type of Matcha.

Following NGS analysis of the Matcha-extracted solution, 12,114,873, 7,590,204, and 10,405,857 clean reads were obtained from MIX, OM, and SM, respectively (Table 1). After searching the public miRNA database (miRBase, https://mirbase.org/) for clean reads, 360, 1230, and 1209 read counts per million (CPM), which is an index for estimating the relative abundance and expression of miRNAs, were annotated as miRNAs, and 390, 504, and 568 miRNA types were identified in MIX, OM, and SM, respectively (Table S1).

**Table 1:** Comprehensive Analysis of miRNAs in Matcha.

Cultivar	Clean Reads	miRNA	
		Mapped Reads	Normalized Reads (CPM)
MIX	12,114,873	4363	360
OM	7,590,204	9338	1230
SM	10,405,857	12,578	1209

The result of NGS analysis of miRNA in three types of Matcha, including blended Matcha (MIX), spring-harvested single cultivar called “Okumidori (OM),” and “Samidori (SM).” The miRNAs were extracted and analyzed using NGS. Counts per million (CPM), an index used to estimate the relative abundance of miRNAs in each sample, was calculated as follows: normalized read counts (CPM) = mapped reads/clean reads × 1,000,000.

**Fig. 1:**
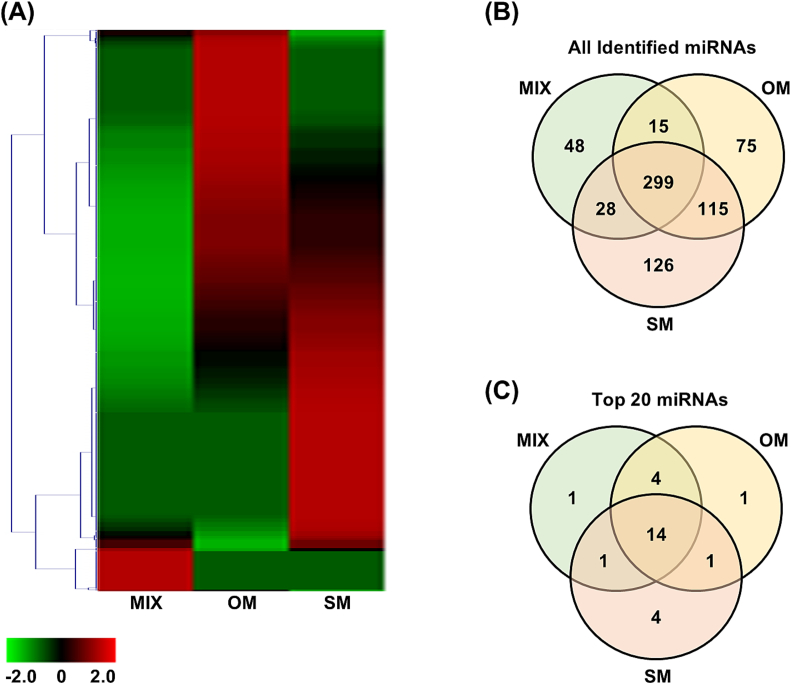
Analysis of Matcha-Derived miRNAs Identified by NGS. miRNAs from three types of matcha including a blended matcha (MIX) and two spring-harvested single cultivar matcha, Okumidori (OM) and Samidori (SM), were extracted and analyzed using NGS. (A) Heatmap and (B) Venn diagram of all identified miRNAs were generated. (C) A Venn diagram of the top 20 ranked miRNAs from three matcha types was generated.

Subsequently, heatmaps and Venn diagrams were generated to investigate the overall patterns of miRNAs in the three different types of Matcha. The heatmap revealed distinct miRNA patterns in the three samples (Fig. 1A). The Venn diagram of the miRNAs identified in this study indicated that among the 706 total miRNAs in the three types of Matcha, 42.4 % (299 types of miRNAs) were common across the three types, 22.4 % (158 types) were shared between only two types of Matcha, and 35.3 % (249 types) were unique to a single Matcha type (Fig. 1B). Both the heatmap and Venn diagram demonstrated the distinct characteristics and expression patterns of miRNAs across the three different Matcha types, indicating that miRNA levels may vary depending on the cultivar. Intriguingly, in the top 20 ranked miRNAs, similar patterns were observed across all three types of Matcha, whereas the ranking of these miRNAs showed minor variations (Table 2). The Venn diagrams of the top 20 ranked miRNAs showed that among the 26 types of miRNAs, 53.8 % (14 types of miRNAs) were common across the three types and 76.9 % were present in at least two types of Matcha (Fig. 1C). Despite slight differences in ranking, the highly expressed miRNAs were generally consistent across the three Matcha types.

**Table 2:** Expression Profiles of miRNAs in Matcha (Top 20).

MIX			OM			SM		
Rank	miRNAs	Normalized Reads (CPM)	Rank	miRNAs	Normalized Reads (CPM)	Rank	miRNAs	Normalized Reads (CPM)
1	lja-miR166-3p	186.8	1	lja-miR166-3p	608.7	1	lja-miR166-3p	576.6
2	csn-miR166c-3p	6.9	2	csn-miRn372	24.0	2	csn-miR396d-5p	32.1
3	csn-miR396d-5p	6.1	3	csn-miR396d-5p	22.4	3	csi-miR166a-3p	15.5
4	csi-miR166a-3p	4.8	4	csn-miR166c-3p	20.7	4	lja-miR168-5p	14.7
5	gma-miR396e	4.5	5	csi-miR166a-3p	14.4	5	csn-miRn372	13.5
6	ahy-miR159	4.1	6	ahy-miR159	13.3	6	osa-miR166g-3p	13.1
7	csn-miRn372	4.0	7	gma-miR396e	12.3	7	csn-miR166c-3p	12.8
8	lja-miR168-5p	3.6	8	csn-miRn409	11.9	8	csn-miRn107	12.5
9	osa-miR166g-3p	3.4	8	csn-miRn431	11.9	9	csn-miRn431	11.3
10	csn-miRn409	3.0	10	lja-miR168-5p	11.1	10	gma-miR156f	10.9
11	csn-miRn230	2.8	11	csn-miRn58	10.8	11	csn-miRn409	9.6
12	csn-miRn343	2.6	12	aqc-miR166a	10.7	12	csn-miRn58	9.5
12	csn-miRn323	2.6	13	csn-miRn31	9.6	13	smo-miR408	9.3
14	csn-miRn431	2.5	14	csn-miRn343	9.2	14	csn-miRn116-3p	9.1
15	csn-miRn469	2.4	15	csn-miRn469	9.1	15	ahy-miR159	8.7
16	csn-miRn58	2.3	16	csn-miRn116-3p	8.8	16	csn-miRn343	8.6
16	csn-miRn31	2.3	17	csn-miRn136	8.4	17	csn-miRn469	8.6
16	csn-miRn315	2.3	18	csn-miRn230	7.9	18	csn-miRn408	7.7
19	csn-miRn116-3P	2.0	19	csn-miRn315	7.8	19	csn-miRn323	7.6
20	csn-miRn180-3P	1.8	20	csn-miRn408	7.2	20	csn-miR166b	7.0

The top 20 miRNAs in the three types of Matcha, including blended Matcha (MIX), spring-harvested single cultivar Okumidori (OM), and Samidori (SM), were identified using NGS analysis.

These results demonstrate the existence of various miRNAs in Matcha. Similar patterns of highly expressed miRNAs were observed across the three different types of Matcha. In contrast, overall patterns revealed characteristic miRNA patterns, indicating that Matcha cultivars might affect the miRNA expression profiles.

### 3.2 Evaluation of miRNA levels in various types of matcha with different cultivars and harvesting seasons

The levels of bioactive components in green teas differ depending on the cultivar used (Fujimura et al., 2022; C. Zhang et al., 2018). Similarly, our NGS results revealed distinct miRNA expression patterns in the three Matcha types. Thus, we hypothesized that miRNAs in Matcha may be present at different levels, potentially leading to varying levels of bioactivity. In addition to the two spring-harvested Matcha cultivars used for NGS analysis, OM and SM, the sample set was expanded to include one additional Matcha cultivar harvested in spring, Saemidori (SMD), and seven more cultivars harvested in the autumn-winter season, including OM, SMD, Yabukita (YB), Yutakamidori (YM), Sayamakaori (SK), Kurasawa (KS), and Zairai (ZR). These Matcha cultivars were selected because they are widely cultivated and consumed in Japan.

**Fig. 2:**
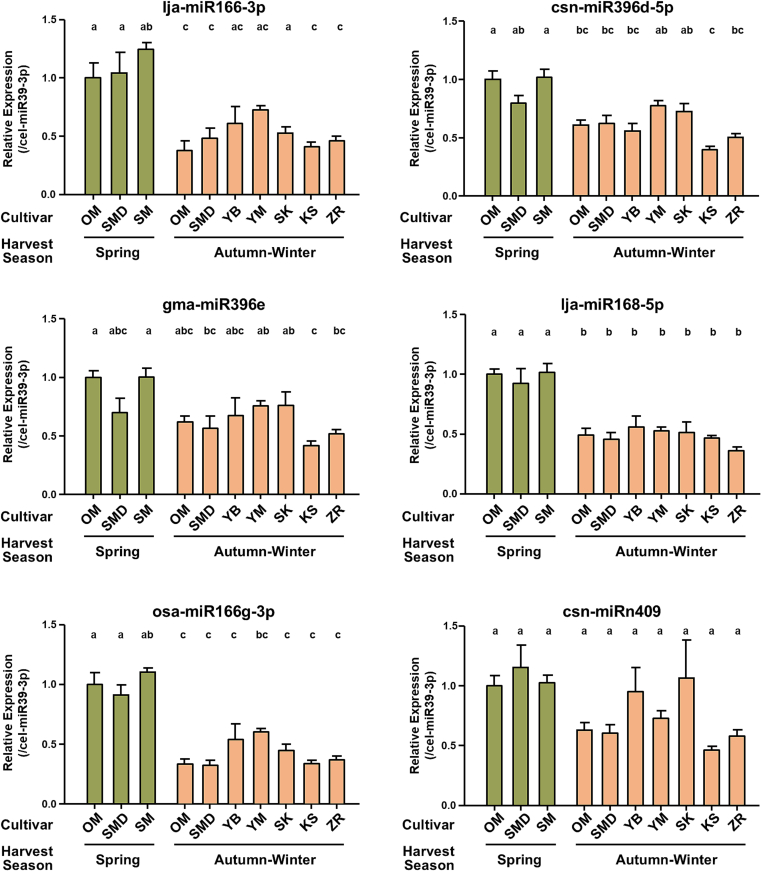
Analysis of Representative Matcha-Derived miRNAs Levels Identified by NGS. Matcha samples were prepared by suspending matcha powder with 80 °C water, followed by the extraction of RNA using Tri Reagent. qRT-PCR analysis of miRNA levels in various matcha samples with different cultivars and harvest season was performed. Data were normalized by cel-miR39-3p (spike in control), and the values were expressed relative to OM. Tukey's Multiple Comparison Test. Data are shown as means ± S.E.M. (*n* = 3–4).

For qRT-PCR analysis, we selected six representative miRNAs with relatively high expression levels based on NGS analysis: lja-miR166-3p, csn-miR396d-5p, gma-miR396e, lja-miR168-5p, osa-miR166g-3p, and csn-miRn409. The results of the qRT-PCR analysis confirmed that these miRNAs were detected in all Matcha samples used in this study (Fig. 2), consistent with the NGS results. Analysis of the 10 Matcha samples suggested that the miRNA levels in Matcha tended to differ depending on the cultivar, as expected, although there were no significant differences.

**Fig. 3:**
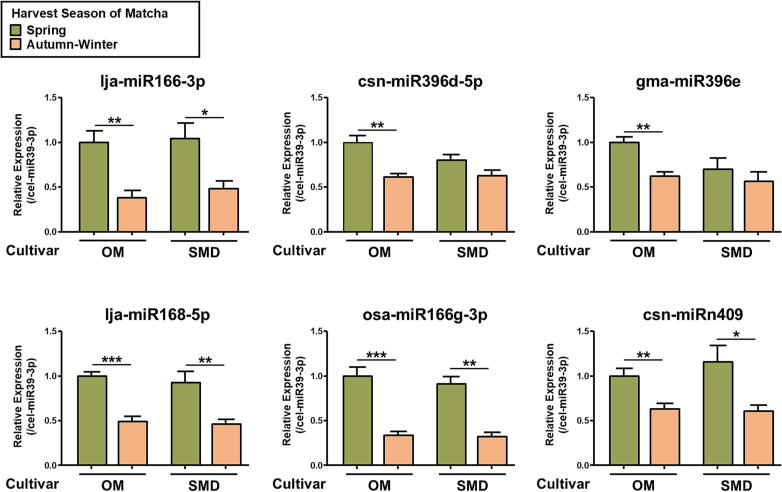
Effects of Harvest Season on the Levels of Representative miRNAs in Matcha. RNA was extracted from matcha suspension solution by Tri Reagent. The levels of six representative miRNA identified by NGS in matcha cultivar “OM” and “SMD” with different harvest seasons were analyzed by qRT-PCR. Data were normalized by cel-miR39-3p (spike in control), and the values were expressed relative to OM. Data are shown as means ± S.E.M. (*n* = 4). Student's *t*-test, *P < 0.05, **P < 0.01, ***P < 0.001.

To further evaluate the effects of environmental factors on miRNAs, we focused on two cultivars, OM and SMD, which were harvested in the spring or autumn-winter seasons. Matcha harvested in spring (spring Matcha) is typically regarded as having the highest quality, characterized by a bright, vibrant green color and rich umami taste. In contrast, Matcha harvested in autumn and winter (autumn-winter Matcha) is generally more robust and slightly astringent, with a deeper, more earthy flavor. The qRT-PCR analysis of the six representative miRNAs revealed significantly higher levels of all six miRNAs in the spring-harvested OM cultivar (Fig. 3). In the SMD cultivar, four miRNAs exhibited significantly higher levels in spring Matcha, whereas a trend of higher levels was observed for the other two miRNAs. These results indicate that miRNA levels differed depending on the Matcha harvesting season.

Our results validated the presence of miRNAs in Matcha by qRT-PCR analysis. Moreover, we observed differences in miRNA levels across ten different types of Matcha, indicating that cultivars and harvesting seasons may be factors that affect miRNA levels.

### 3.3 Evaluating the RNA extraction methods for miRNA analysis of matcha

**Fig. 4:**
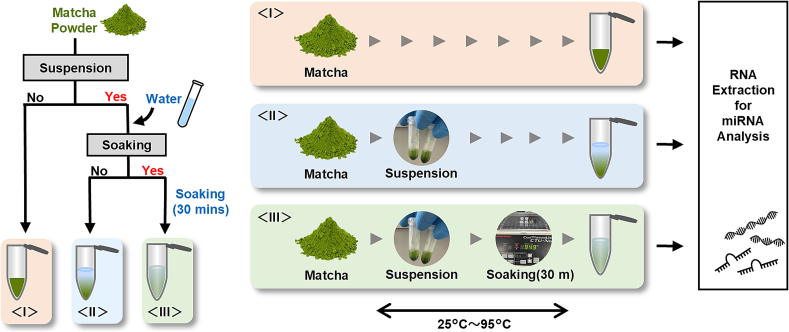
Overall Experimental Design to Evaluate the Extraction Methods of RNA in Matcha for miRNA Analysis. Matcha sample (OM harvested in spring) were prepared under three different conditions: < I > no treatment, < II > suspending with water at temperatures ranging from 25 °C to 95 °C without soaking, and < III > suspending with water at temperatures ranging from 25 °C to 95 °C with 30 min of soaking at each temperature. Following the treatment, RNA was extracted for further analysis.

Efficient methods for extracting miRNAs from Matcha remain largely unknown, particularly for functional studies. Here, we aimed to evaluate the effects of several common factors on the RNA extraction efficiency for miRNA analysis. Considering the typical and traditional conditions under which Matcha is consumed as a hot water infusion, we utilized OM cultivar and focused on two factors: hot water suspension ranging from 25 °C to 95 °C (Fig. 4, group < II >) and the process of soaking Matcha under the same temperature conditions (Fig. 4, group < III >). Additionally, as Matcha powder is used directly as an additive in cakes, ice cream, and cookies, we included a control group in which miRNAs were extracted directly from Matcha powder without additional treatment (Fig. 4, group < I >).

**Fig. 5:**
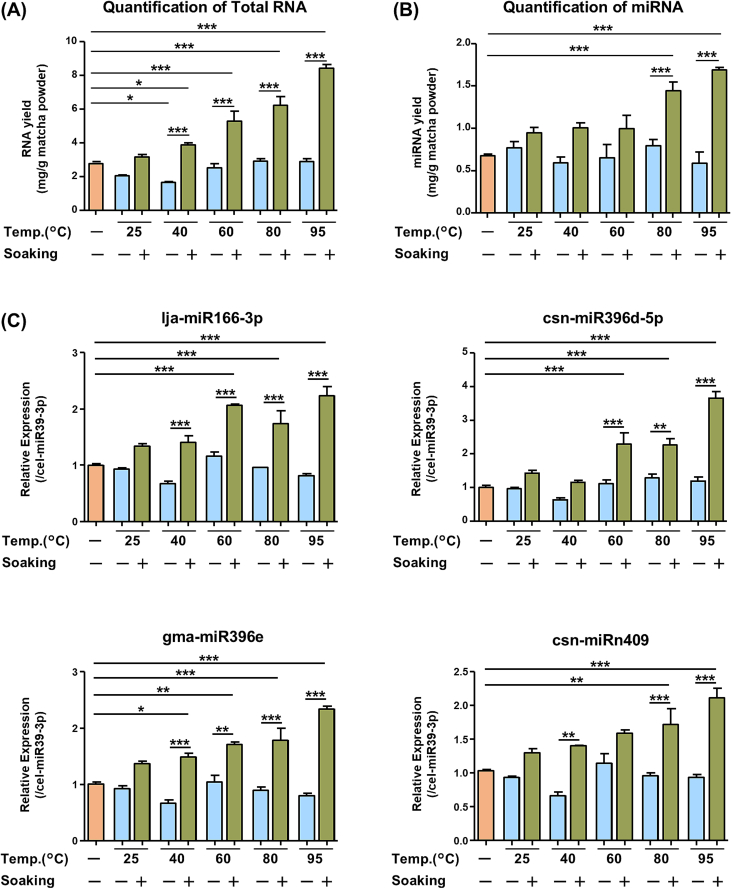
Evaluation of Extraction Methods of RNA in Matcha for miRNA Analysis. Matcha samples were prepared by different treatments, and (A) RNA yield and (B) miRNA yields were measured by Nanodrop and Qubit microRNA Assay Kits, respectively. (C) qRT-PCR analysis of four representative miRNA levels in each samples were performed. Data were normalized by cel-miR39-3p (spike in control) and multiplied by dilution factor when diluting RNA for cDNA synthesis to obtain the original expression levels of miRNA in each sample. The values were expressed relative to the group < I > and shown as means ± S.E.M. (*n* = 3–4). Tukey's Multiple Comparison Test, *P < 0.05, **P < 0.01, ***P < 0.001. (Significant differences are indicated only for comparisons with group < I > and within the same temperature group (group < II > and < III >)).

Under these RNA extraction conditions, we found that suspending Matcha powder in water at temperatures between 25 °C and 95 °C (group < II >) did not significantly enhance RNA extraction compared with direct extraction from Matcha powder (group < I >) (Fig. 5A). The lack of significant differences in RNA yield between groups < I > and < II > indicated that simply suspending the Matcha powder in hot water had minimal effect on RNA extraction. However, soaking Matcha powder in hot water (group < III >) resulted in a remarkable increase in RNA yield compared with that in the < I > group. Specifically, soaking at 40 °C, 60 °C, 80 °C, and 95 °C resulted in 1.40-fold, 1.91-fold, 2.26-fold, and 3.05-fold increases of RNA yields, respectively, compared to group < *I* >. These results revealed that soaking Matcha powder at temperatures above 40 °C significantly enhances RNA yield in a temperature-dependent manner, with the highest yield observed at 95 °C.

Conversely, unlike total RNA, a clear temperature-dependent increase in miRNA yield above 40 °C in soaked samples (group < III >) was not observed compared to group < I >. By contrast, increases were still observed in 40 °C and 60 °C-soaked samples (Fig. 5B). From 80 °C and 95 °C, notable increases in miRNA yields were observed in soaked samples, suggesting that utilizing hot water above 80 °C may be able to effectively enhance miRNA yields. To further confirm these findings, we performed qRT-PCR analysis on the four miRNAs that showed the highest expression based on the NGS results: lja-miR166-3p, gma-miR396e, csn-miR396d-5p, and csn-miRn409. Consistent with the results of RNA and miRNA yields, suspending Matcha powder in water at temperatures ranging from 25 °C to 95 °C did not significantly increase miRNA levels compared with group < I > (Fig. 5C). The soaking process enhanced four representative miRNA levels, especially when Matcha powder was soaked at temperature above 60 °C, with the highest miRNA levels observed at 95 °C. Similar to total RNA, the levels of gma-miR396e and csn-miRn409 were enhanced in soaked samples in a temperature-dependent manner, whereas this was not observed for lja-miR166-3p and csn-miR396d-5p, indicating that these conditions might affect the extraction of each miRNA.

Consequently, we demonstrated that utilizing 95 °C hot water for suspension and soaking provided the highest yielding of RNA and miRNA, as well as several representative miRNA levels. The qRT-PCR analysis also revealed that the extraction of each miRNA varied slightly, depending on the extraction conditions. These results provide fundamental information regarding the RNA extraction methodology that supports further functional analysis of miRNAs.

### 3.4 Investigation of miRNAs in various commercially available plant-based powders

Next, we investigated whether the miRNAs identified by NGS were unique to Matcha and evaluated the applicability of the extraction method (Fig. 4, Fig. 5). In addition to Matcha (OM harvested in spring), we collected various plant-based commercially available products that are easily accessible in daily life, including tea (e.g., sencha and oolong tea), dried vegetables (e.g., komatsuna and eggplant), and spices (e.g., black pepper and cinnamon) (Table 3 and Fig. S1). As no studies have attempted to perform RNA extraction of such dried plant-based commercial powder, and whether RNA can be extracted from such samples is questionable, we first attempted to extract RNA under the conditions where 95 °C hot water was added to samples and soaked for 30 min.

**Table 3:** RNA and miRNA Yield of Various Plant-based Dried Powders.

Category	Sample Name	RNA yield (mg/g powder)	miRNA yield (mg/g powder)
Tea	Matcha	10.19 ± 0.34	1.95 ± 0.04
	Gyokuro	4.62 ± 0.41	1.39 ± 0.06
	Fukamushicha (Deep-Steamed Sencha)	4.00 ± 0.25	0.81 ± 0.05
	Sencha	4.00 ± 0.11	1.29 ± 0.03
	Kukicha (Stalk Green Tea)	1.21 ± 0.29	0.83 ± 0.02
	Hojicha (Roasted Green Tea)	2.58 ± 0.05	1.09 ± 0.07
	Bancha (Coarse Green Tea)	1.21 ± 0.06	0.50 ± 0.05
	Tamaryokucha	2.72 ± 0.07	0.73 ± 0.06
	Oolong Tea	1.74 ± 0.01	0.64 ± 0.05
Spices	Black Pepper	0.45 ± 0.03	*n.d.*
	Cinnamon	0.36 ± 0.13	*n.d.*
	Sage	0.37 ± 0.21	*n.d.*
	Thyme	0.36 ± 0.04	*n.d.*
	Parsley	1.24 ± 0.07	0.93 ± 0.02
	Basil	2.24 ± 0.13	1.65 ± 0.02
	Oregano	0.39 ± 0.00	*n.d.*
	Cilantro	4.69 ± 0.10	2.35 ± 0.02
	Rosemary	0.11 ± 0.06	*n.d.*
	Cardamom	0.36 ± 0.08	*n.d.*
	Coriander	0.73 ± 0.04	*n.d.*
Dried Vegetables	Komatsuna (Japanese Mustard Spinach)	2.96 ± 0.28	1.83 ± 0.01
	Spinach	1.11 ± 0.07	0.72 ± 0.01
	Eggplant	0.85 ± 0.05	0.19 ± 0.00
Others	Ground Sesame	0.62 ± 0.09	0.54 ± 0.02
	Wakame (Japanese Seaweed)	0.84 ± 0.07	0.59 ± 0.03
	Kinako (Roasted Soybean Flour)	3.91 ± 0.19	2.43 ± 0.03
	Green Laver	4.15 ± 0.26	2.03 ± 0.05

Each sample was suspended in 95 °C water and soaked for 30 min at 95 °C. Following treatment, the RNA and miRNA yields were measured using Nanodrop and Qubit microRNA Assay Kits, respectively. For the species samples, only those with RNA yields exceeding 1.00 mg/g of powder were further analyzed for miRNA (*n.d.* indicates samples yielding less than 1.00 mg/g RNA). Data are shown as means ± S.E.M. (*n* = 3).

**Fig. 6:**
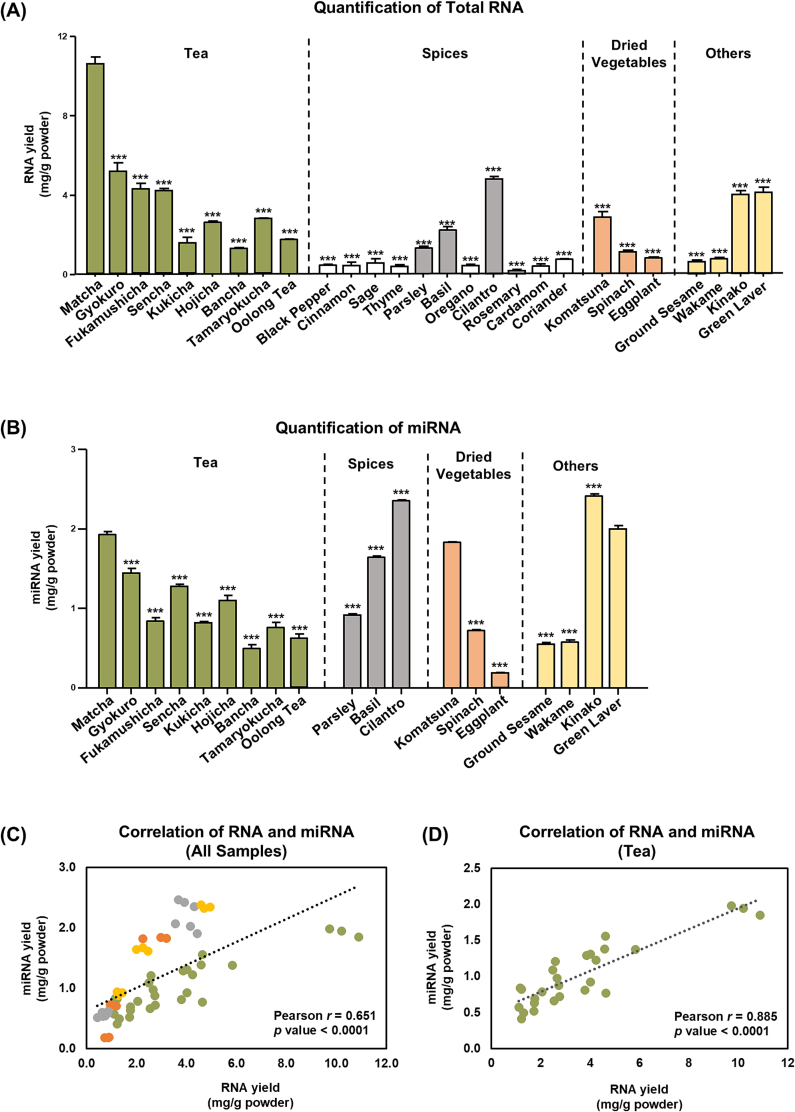
Yielding of RNA and miRNA in Various Plant-Based Powders. Samples were prepared by suspending with 95 °C water at soaked for 30 mins at 95 °C. (A) RNA yield and (B) miRNA yields were measured. For the species samples, only those with RNA yields exceeding 1.00 mg/g of powder were further analyzed for miRNA. (C–D) Pearson correlation analysis of RNA and miRNA yield were conducted. Data are shown as means ± S.E.M. (*n* = 3). Student's *t*-test vs Matcha, *P < 0.05, **P < 0.01, ***P < 0.001.

Under these conditions, RNA yield differed greatly depending on the sample type. For 1 g of the sample, RNA yields ranged from 1.21 mg to 10.19 mg for tea, 0.11 mg to 4.69 mg for spices, 0.85 mg to 2.96 mg for dried vegetables, and 0.62 mg to 4.15 mg for the other samples. Among all the samples tested, those in the tea category generally yielded the highest amounts of RNA. Within the tea category, Matcha had the highest RNA yield. For the measurement of miRNA yield, all samples were analyzed, except those with RNA yields of less than 1 mg/g in the category of spices. miRNA yields for 1 g of the sample ranged from 0.50 mg to 1.95 mg for tea, 0.93 mg to 2.35 mg for spices, 0.19 mg to 1.83 mg for dried vegetables, and 0.54 mg to 2.43 mg for the other samples (Fig. 6B). Although Matcha did not have the highest miRNA yield among all samples, it exhibited the highest yield among the nine tea types prepared using different processing and cultivation methods.

Although correlations between individual mRNAs and specific miRNAs have been documented (Ruike et al., 2008), little is known about the relationship between the total RNA and miRNA yields. In our study, we observed a moderate positive correlation (Pearson *r* = 0.651, *p* < 0.0001) between RNA and miRNA yields across all sample types (Fig. 6C). In tea samples, a strong positive correlation (Pearson *r* = 0.885, *p* < 0.0001) was observed between RNA and miRNA yields (Fig. 6D). This correlation implies that the quantification of total RNA may serve as a convenient and cost-effective preliminary screening method for estimating miRNA levels by reducing the need for more complex and costly miRNA-specific quantification methods.

**Fig. 7:**
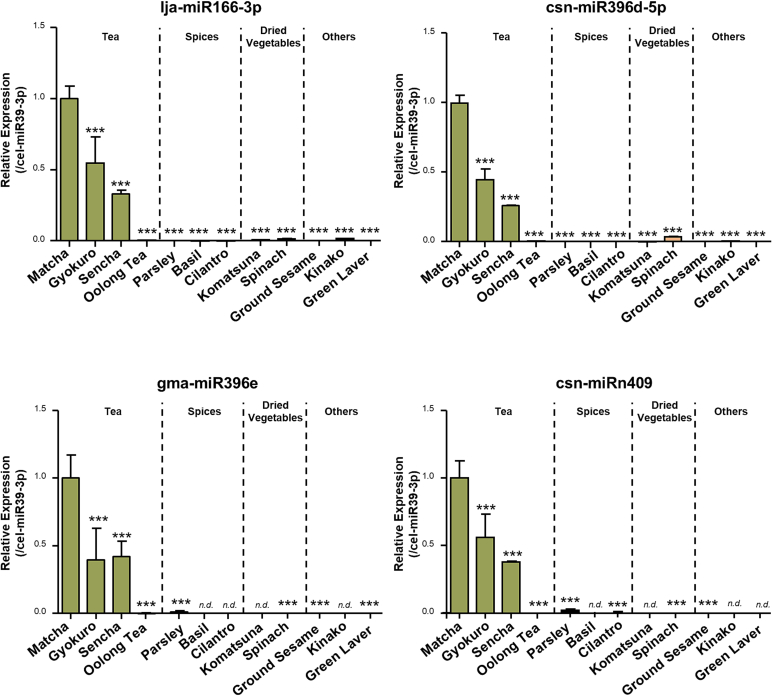
Analysis of miRNA in Various Plant-Based Powders. Samples were prepared by suspension with 95 °C water and 30 mins soaking at 95 °C and qRT-PCR analysis of four representative miRNA levels was performed. Data were normalized by cel-miR39-3p (spike in control) and multiplied by dilution factor when diluting RNA for cDNA synthesis to obtain the original expression levels of miRNA in each samples. The values were expressed relative to matcha group. Data are shown as means ± S.E.M. (*n* = 3). Dunnett's comparison test vs Matcha, *P < 0.05, **P < 0.01, ***P < 0.001. *n.d.* indicated not detection.

To examine whether the miRNAs identified by NGS were unique to Matcha, we selected representative samples from each category and performed qRT-PCR analysis of the four representative miRNAs that were highly expressed in Matcha. Sencha and gyokuro were chosen because sencha is the most consumed type of green tea and gyokuro yielded the second-most RNA. Oolong tea was selected from the tea category because of its distinct processing methods and degree of fermentation. For the other categories, samples were selected based on their miRNA yields. The qRT-PCR results revealed that lja-miR166-3p, csn-miR396d-5p, gma-miR396e, and csn-miRn409 were mainly detected in the green tea samples, including Matcha, gyokuro, and sencha, with Matcha exhibiting the highest levels of miRNAs (Fig. 7). In contrast, in oolong tea, these four miRNAs were detectable but at extremely low levels. Specifically, the levels of these four representative miRNAs in oolong tea were less than 0.5 % of those in Matcha.

Our results demonstrated that, among the 27 types of commercially available plant-based powders, Matcha exhibited the highest RNA yields and the highest levels of the four representative miRNAs, implying the potential of Matcha as a rich source of miRNAs. Additionally, the total RNA and miRNA yields were positively correlated across all samples. These results provide basic information regarding the miRNA properties of various plant-based powders that are often consumed daily and should contribute to further functional analyses.

### 3.5 Correlation analysis between miRNA yields and major components in matcha

**Fig. 8:**
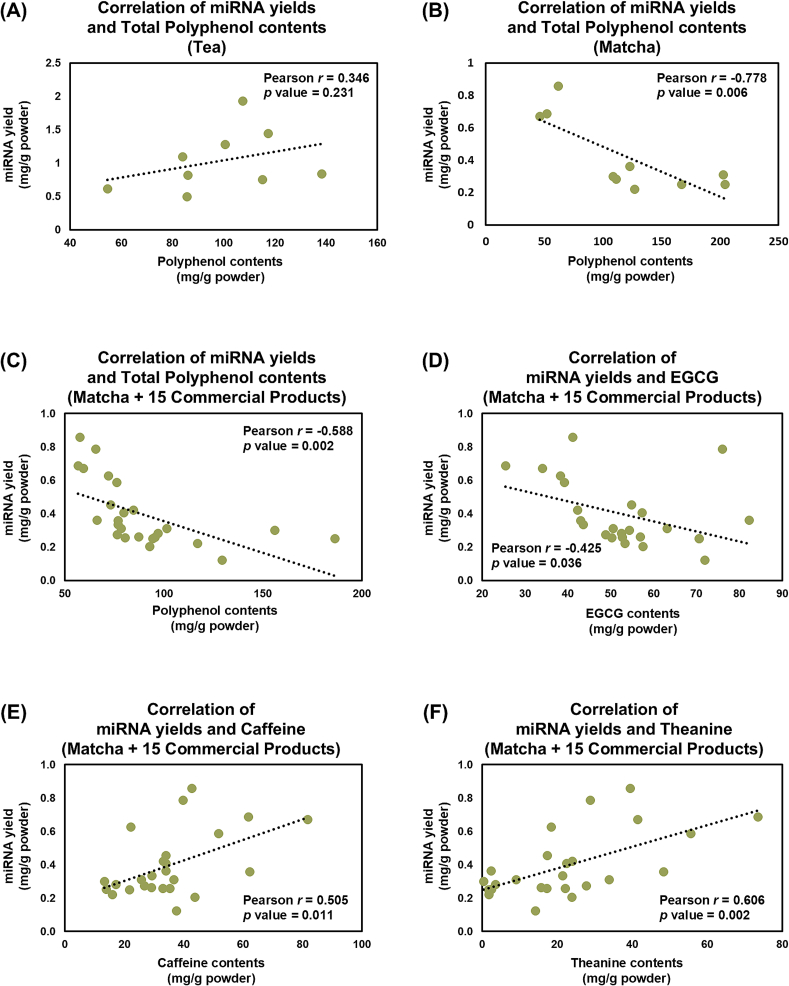
Correlation Analysis of miRNA and Polyphenol Contents. Pearson correlation analysis between miRNA yields and total polyphenol contents were conducted (A) across all tea samples (tea samples in Table 3) and (B) only in matcha samples (10 samples used in Fig. 2, Fig. 3). In additional to the matcha samples, we conducted further analysis by including 15 more commercially available matcha products. LC-MS analysis of EGCG, caffeine and theanine levels was conducted and Pearson correlation analysis between miRNA yield and (C) total polyphenol amounts, (D) EGCG, (E) caffeine or (F) theanine levels was performed. Data are shown as average values (*n* = 3).

Polyphenols have attracted significant attention in functional studies on green tea as major bioactive compounds (Kumazoe & Tachibana, 2016; Maeda-Yamamoto & Tachibana, 2020; Mokra et al., 2022; Musial et al., 2020; Tachibana, 2011; Umeda et al., 2008). However, the relationship between polyphenol content and miRNA yield in Matcha has not yet been documented. Thus, in this study, we first analyzed the correlation between the total polyphenol content and miRNA yield. No correlation (Pearson *r* = 0.346, *p* = 0.231) was observed across all tea samples, including the 9 types of tea, such as Matcha, sencha, gyokuro, and oolong tea used in the previous experiment (Fig. 8A). Considering that most of these teas underwent different degrees of processing, such as steaming and oxidation, we conducted a correlation analysis using only the Matcha samples. A significant negative correlation (Pearson *r* = −0.778, *p* = 0.006) was observed among the 10 types of Matcha samples with different cultivars and harvesting seasons used in the previous experiment (Fig. 8B). Furthermore, we examined whether this correlation between polyphenol contents and miRNA yield exists in Matcha powders that are commercially sold in Japan. A moderate negative correlation was observed (Pearson *r* = −0.588 with *p* value = 0.002) in 25 Matcha powders including 10 samples used in previous experiments and 15 commercially available products.

Eigallocatechin-3-*O*-gallate (EGCG) and caffeine have been extensively studied for their physiological effects. Thus, we conducted a correlation analysis between miRNA yields and EGCG or caffeine levels in 25 Matcha powders. Similar to the negative correlation observed with polyphenol, miRNA yields were negatively correlated with EGCG amount (Fig. 8D, Pearson *r* = −0.425 with *p* value = 0.036). In contrast, a positive correlation was observed between miRNA yields and caffeine (Fig. 8E, Pearson *r* = 0.505, *p* = 0.011). Moreover, theanine, another major component attributed to the functionality of Matcha, also exhibited a positive correlation with miRNA yield (Fig. 8F, Pearson *r* = 0.606, *p* = 0.002).

These results demonstrate a relationship between miRNA yields and several major components in Matcha, including negative correlations with total polyphenol contents and EGCG, and positive correlations with caffeine and theanine. These results revealed the basic properties of miRNAs that would be helpful when considering the functionality of Matcha.

## 4 Discussion

Several recent studies have highlighted the beneficial effects of plant miRNAs on animals, suggesting the potential of food-derived plant miRNAs to exert various biological functions through cross-kingdom regulation (Chen et al., 2020; Kumazoe et al., 2023; Liu et al., 2021; Samad et al., 2021; L. Zhang et al., 2019). In the present study, we revealed, for the first time, the existence of miRNAs in Matcha, a traditional powdered Japanese green tea that is well-known for its health benefits. While previous studies on miRNAs in tea plants primarily used fresh leaves and focused on environmental stress responses (Jeyaraj et al., 2017; Zheng et al., 2015), our study demonstrated that diverse miRNAs remained intact, even after Matcha manufacturing. The discovery of these miRNAs in Matcha has introduced a novel class of molecules with great potential to exert physiological effects. These results contribute to food functionality research, particularly in understanding bioactive components beyond the major components, including polyphenols and caffeine.

The presence of miRNAs as novel food components in Matcha powder prompted us to investigate whether miRNAs exist in other plant-based powders. Therefore, in addition to Matcha powders, this study included a wide range of plant-based powdered products that are generally available in Japan, such as teas, spices, and dried vegetables, for miRNA analysis. While specific miRNAs have been reported in some samples, such as spices, to the best of our knowledge (Sarkar et al., 2024), there are currently no data comparing total RNA or miRNA yields from these samples. Results comparing the miRNA yields should provide fundamental information that supports future functional research on plant-derived miRNAs. We also found that spices, plant substances that have attracted attention for their ability to protect against the development of acute and chronic non-communicable diseases (Jiang, 2019), exhibited high miRNA yields. This indicated the potential of miRNAs as candidate functional factors in this species. Among all analyzed samples, the tea samples yielded the highest amount of RNA. Matcha exhibited the highest RNA yield and highest levels of the four representative miRNAs among the tea samples. These findings indicate the potential of Matcha as a rich source of miRNAs that may be suitable for the functional analysis of miRNAs.

Dietary miRNAs were recently shown to exert physiological effects in animals, as first reported by Zhang et al. Plant miR168a decreases low-density lipoprotein removal from mouse plasma by targeting LDLRAP1 (L. Zhang et al., 2011). Another study demonstrated that miR172d-5p, a plant miRNA derived from rice (*Oryza sativa*), suppressed the expression of fibrosis-related genes, thereby exhibiting an anti-fibrotic effect, as confirmed by in vivo experiments (Kumazoe et al., 2023). Together with these studies, the cross-kingdom regulation of dietary miRNAs has recently attracted attention (Samad et al., 2021; L. Zhang et al., 2019). Notably, ath-miR159a, a miRNA detected in Matcha, has been previously reported to inhibit breast cancer growth both in vitro and in vivo (Liu et al., 2021), implying the potential of Matcha-derived miRNAs to exert health-promoting effects. Although little is known about the functions of most Matcha-derived miRNAs, including the four representative miRNAs analyzed in this study, these results provide information regarding the miRNAs in Matcha and may be useful for accelerating further functional research on Matcha.

Along with functional studies on plant miRNAs, research on their practical applications has progressed in recent years (Xie & Melzig, 2018). Matcha is available in various forms, including hot water infusions and intact dried powder. Hot water infusion is typically used in traditional ceremonies or directly consumed as a beverage in daily life. Powdered Matcha is often used to produce various snacks and food products. Previous studies have shown that the extraction of bioactive components from green tea, such as catechins, caffeine, and amino acids, is significantly affected by the extraction conditions (Allameh & Orsat, 2023; Jin et al., 2019). However, the extraction of miRNAs from Matcha remains unclear. Our results suggest that increasing the water temperature during the soaking process enhances miRNA yields. Enhanced miRNA extraction at high temperatures may result from cell membrane deformation and damage, facilitating solvent permeability through plant tissues (Dias et al., 2020; Lima et al., 2013). Without soaking process, increasing the temperature of hot water did not enhance the miRNA yield. Performing RNA extraction immediately after the addition of hot water may not allow enough time for the disruption of plant cell membranes to facilitate the release of miRNAs. Moreover, the extraction of bioactive components, including catechins and caffeine, was enhanced at higher temperatures in hot water (Allameh & Orsat, 2023; Jin et al., 2019). miRNAs exhibit characteristics similar to those of the major functional components of Matcha, such as catechins and caffeine. These findings suggest a basic relationship between miRNAs and their major functional components in Matcha. Our findings may be useful when considering consumption methods that allow more functional components to be consumed to enhance the health-promoting potential of Matcha. Furthermore, our results demonstrated that soaking Matcha in hot water above 40 °C enhanced the levels of representative miRNAs. This finding is particularly noteworthy, given that the traditional way to consume Matcha often involves the use of hot water above 60 °C, allowing Matcha-derived miRNAs to be easily consumed without any other special preparation steps.

Different cultivars and harvest seasons of green tea are generally known to exhibit variations in composition, including polyphenols and caffeine (Fujimura et al., 2022; C. Zhang et al., 2018). Similarly, we not only demonstrated the existence of various miRNAs in Matcha but also observed that miRNA levels differed depending on the cultivar and harvest season. This finding is consistent with a previous report on rice, in which miRNA expression patterns differed between the two cultivars (Nguyen et al., 2023). Several studies have reported that miRNA levels in plants are affected by numerous environmental factors, such as temperature (Re et al., 2019), light quantity (Islam et al., 2023) and quality (Dong et al., 2020), and nutrient conditions (Islam et al., 2022), which are known to differ among harvest seasons. Lower ambient temperature has been reported to enhance the activity of the plant miRNA biogenesis machinery (Re et al., 2019). This study consistently demonstrated that miRNA yields of Matcha harvested in spring, which typically grow under lower-temperature conditions, were higher than those grown during summer under higher-temperature conditions. In addition to temperature regulation, the amount of sunlight directly regulates miRNA biogenesis by controlling RNA-binding proteins that participate in miRNA processing, such as hyponastic leaves1 (HYL1) and HYL1-cleavage subtilase 1 (HCS1) (Islam et al., 2023). In Japan, sunlight quantity is considered a primary distinguishing factor between spring-harvested and autumn-winter-harvested Matcha because spring-harvested Matcha is often exposed to less intensive sunlight. Although further research on plant physiology is necessary, our results suggest that miRNA yields may be regulated by temperature and sunlight exposure. This information may be helpful when considering cultivation methods and cultivar selection of Matcha to evaluate or obtain functionality related to Matcha-derived miRNAs.

In addition to miRNA biogenesis, light is a key regulator in the biosynthesis of several bioactive components in green tea, including polyphenols (Hong et al., 2014), which have been extensively studied for their physiological roles. However, the relationship between the miRNA yield and polyphenols remains unknown. We observed a negative relationship between the polyphenol content and miRNA yield. In Matcha, EGCG is the major polyphenol that largely contributes to its physiological effects. Despite the negative correlation between the miRNA yield and polyphenol content, EGCG also showed a negative correlation with the miRNA yield. miRNAs have been reported to target several proteins involved in regulating the biosynthesis of catechins (Li et al., 2021; Sun et al., 2017), the most abundant secondary metabolites in tea plants, accounting for 70 % of all polyphenols. Csn-miRn70, one of the miRNAs detected in this study, was reported to regulate the expression of flavonoid 3-hydroxylase (F3H), a key enzyme in the biosynthesis of catechins in tea plants (Sun et al., 2017). In addition, our NGS analysis also revealed the presence of several miRNAs in Matcha, including csn-miRn23, csn-miRn27, csn-miRn49, and csn-miRn56, which were previously suggested to regulate the expression of flavonoid 3′,5′-hydroxylase (F3′5′H), a main enzyme for the formation of catechins (Sun et al., 2017). As the relationship between the levels of the abovementioned miRNAs and catechins was not analyzed in this study, research on this relationship is necessary to understand the functional components of Matcha. Environmental factors, particularly sunlight exposure, regulate polyphenol (Hong et al., 2014) and nucleic acid synthesis (Islam et al., 2023). Consistent with these findings, in Fig. 8B, our correlation analysis showed that spring-harvested Matcha were present in the upper-left corner of the correlation plot (high miRNA yields with low polyphenol contents), and autumn-winter harvested Matcha were present in the lower-right corners (low miRNA yields with high polyphenol contents). Although the underlying mechanisms remain unknown, our results are the first to reveal the relationship between miRNA yield and polyphenol content in Matcha and suggest that sunlight may be a factor regulating these components.

Numerous Matcha studies have investigated health-promoting roles of theanine and caffeine; however, the relationship between miRNAs and these compounds is unknown. In this study, we observed positive correlations between miRNA yield and theanine or caffeine levels in Matcha. The Matcha samples used in the analysis showed a higher accumulation of theanine and caffeine in spring-harvested Matcha, which is consistent with previous studies (Deka et al., 2021; Huang et al., 2022). The nitrogen cycle plays a crucial role in the biosynthesis of theanine (Takeo, 1981; Y. Wang et al., 2021) and caffeine (Cai et al., 2024) in tea plants. During the spring, nitrogen metabolism is highly activated, enhancing the accumulation of theanine and caffeine (Takeo, 1981). In addition, Matcha-oriented tea cultivation often uses large amounts of nitrogen-rich fertilizers, and this nitrogen supply contributes to higher levels of caffeine and theanine in the tea leaves (Qiu et al., 2024). Therefore, the elevated theanine and caffeine levels observed in spring-harvested Matcha may be due to both naturally elevated nitrogen metabolism and exogenous nitrogen supply. Nitrogen is an essential component of the nucleic acid bases of RNA, and nitrogen-related metabolites have been reported to modulate gene expression in plants (Fonseca et al., 2022). In the present study, we demonstrated a positive correlation between RNA and miRNAs. Although direct evidence is currently lacking, our findings suggest that the biosynthesis of RNA, including miRNAs, may also be regulated by nitrogen supply, similar to the accumulation of theanine and caffeine. Overall, the observed correlations between miRNAs and the three major functional components (EGCG, caffeine, and theanine) in Matcha are crucial for understanding the functional factors that contribute to the health benefits of Matcha. Generally, Matcha is sold as a blended product consisting of multiple cultivars. Thus, our findings are important for selecting Matcha cultivars for blending and further promoting their practical applications. Compositional differences can affect bioactivity (Fujimura et al., 2022) and taste (L. Zhang et al., 2020) in green tea. Amino acids, including theanine, are generally abundant in spring-harvested tea and contribute to umami and sweet tastes (Huang et al., 2022; Kaneko et al., 2006). In addition, spring-harvested Matcha had a higher caffeine concentration, which contributed to the bitter taste of the Matcha, while autumn-winter harvested Matcha had a higher catechin concentration, which contributed to its astringency (Deka et al., 2021; L. Zhang et al., 2020). However, nothing is known about the effects of miRNAs on the taste profiles of Matcha. Therefore, unraveling the potential relationship between miRNAs and taste may lead to a better understanding of the fundamental properties of Matcha.

## 5 Conclusion

Collectively, this study revealed, for the first time, the presence of functional miRNAs in Matcha and that the expression levels of representative miRNAs are affected by the harvest season and cultivar. Compared with 27 commercially available plant-based dried powders, Matcha exhibited the highest RNA yields and levels of the four representative miRNAs, indicating that Matcha is a rich miRNA source. An investigation of RNA extraction methods to ensure efficient miRNA yield revealed that miRNA yields were enhanced by increasing the hot water temperature during soaking. Furthermore, our results demonstrated that miRNA yields were negatively correlated with polyphenol and EGCG content and positively correlated with theanine and caffeine levels. Although functional evaluation of Matcha miRNAs is required, such compositional information may provide new insights into the understanding of health-promoting effects of Matcha. These findings may be useful for the selection of Matcha cultivars and consumption methods that allow the consumption of more functional components.

## CRediT authorship contribution statement

**Yi-Lan Huang:** Writing – original draft, Methodology, Investigation, Formal analysis. **Tomomi Morikawa-Ichinose:** Writing – review & editing, Methodology, Investigation, Funding acquisition, Formal analysis, Conceptualization. **Seong-Uk Lee:** Methodology, Investigation, Formal analysis. **Yuka Tatsumi:** Methodology, Investigation, Formal analysis. **Masaki Ichitani:** Methodology, Investigation, Formal analysis. **Motofumi Kumazoe:** Methodology, Investigation, Formal analysis. **Hirofumi Tachibana:** Writing – review & editing, Supervision, Funding acquisition, Conceptualization. **Yoshinori Fujimura:** Writing – review & editing, Supervision, Methodology, Investigation, Funding acquisition, Formal analysis, Conceptualization.

## Funding information

This study was supported in part by Matcha and Health Research (to Y.F.) and JSPS KAKENHI, Grant Numbers JP22K05525 (to T.M.I), JP20H05683 (H.T.), JP23K26856, and JP24K21841 (Y.F.).

## Declaration of competing interest

The authors declare that they have no known competing financial interests or personal relationships that could have appeared to influence the work reported in this paper.

## Data Availability

Data will be made available on request.

## Appendix A Supplementary data

Supplementary material 1 Image 1

Supplementary material 2 Image 2
