# Supplementary material for: Comprehensive microRNA analysis toward exploring a new functional component in Matcha green tea
Source: Food Chem (Oxf). 2025 May 28;10:100265. doi: 10.1016/j.fochms.2025.100265 (PMC12171767; doi:10.1016/j.fochms.2025.100265)
Supplement: Supplementary file 1 — Supplementary material 1 [file mmc1.docx]

**Supplementary Material**

**Comprehensive MicroRNA Analysis Toward Exploring a New Functional Component in Matcha Green Tea**

Yi-Lan Huang^1,#^, Tomomi Morikawa-Ichinose^1,#^, Seong-Uk Lee^1^, Yuka Tatsumi^2^, Masaki Ichitani^2^, Motofumi Kumazoe^1^, Hirofumi Tachibana^1^, Yoshinori Fujimura^1,^*****

^1^Division of Applied Biological Chemistry, Department of Bioscience and Biotechnology, Faculty of Agriculture, Kyushu University, 744 Motooka, Nishi-ku, Fukuoka 819-0395, Japan

^2^Central Research Institute, ITOEN, Ltd., 21 Mekami, Makinohara-shi, Shizuoka 421-0516, Japan

^#^These authors contributed equally to this work.

***Corresponding author:** Yoshinori Fujimura, PhD

**Present address**

Division of Applied Biological Chemistry, Department of Bioscience and Biotechnology, Faculty of Agriculture, Kyushu University, 744 Motooka, Nishi-ku, Fukuoka 819-0395, Japan

Tel: 81-92-802-4747 / Fax: 81-92-802-4748

E-mail address: [fujimu@agr.kyushu-u.ac.jp](mailto:fujimu@agr.kyushu-u.ac.jp)


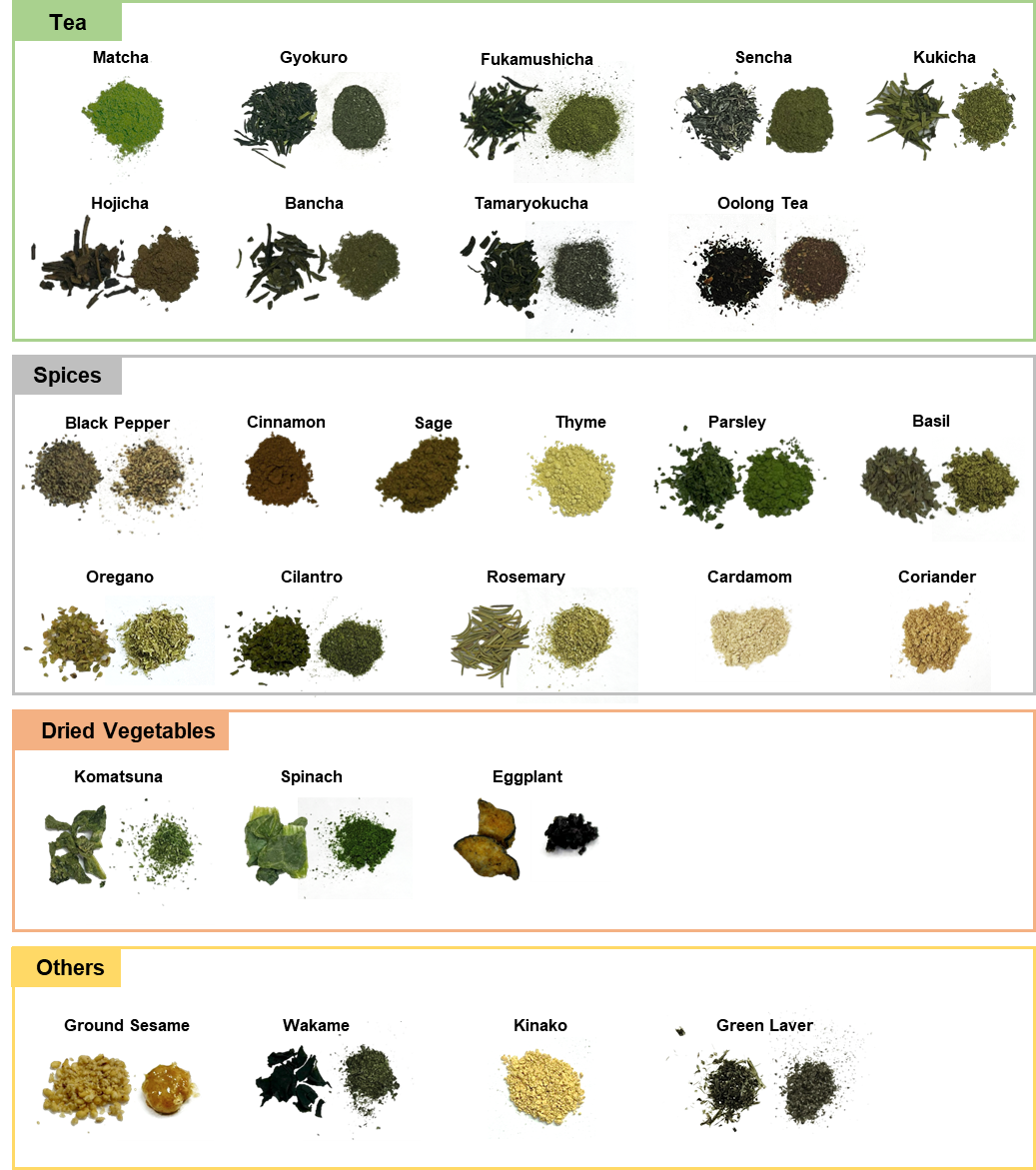


**Fig. S1 Commercially Available Plant-Based Dried Powders**

miRNAs in various commercially available plant-based dried powders were analyzed. Samples originally in powder form were displayed as single images. Samples in the non-powdered form were shown with images of the original form (left) and the powdered form (right). Non-powdered samples were homogenized using stainless steel beads with a micro smash instrument, as mentioned in the section of 2.1 Materials.
